# Proteome balancing of the maize seed for higher nutritional value

**DOI:** 10.3389/fpls.2014.00240

**Published:** 2014-05-30

**Authors:** Yongrui Wu, Joachim Messing

**Affiliations:** ^1^Institute of Plant Physiology and Ecology, Shanghai Institutes for Biological Sciences, Chinese Academy of SciencesShanghai, China; ^2^Waksman Institute of Microbiology, Rutgers UniversityPiscataway, NJ, USA

**Keywords:** nitrogen, lysine, sulfur, storage proteins, RNAi

## Abstract

Most flowering plant seeds are composed of the embryo and endosperm, which are surrounded by maternal tissue, in particular the seed coat. Whereas the embryo is the dormant progeny, the endosperm is a terminal organ for storage of sugars and amino acids in proteins and carbohydrates, respectively. Produced in maternal leaves during photosynthesis, sugars, and amino acids are transported to developing seeds after flowering, and during germination they nourish early seedlings growth. Maize endosperm usually contains around 10% protein and 70% starch, and their composition ratio is rather stable, because it is strictly regulated through a pre-set genetic program that is woven by networks of many interacting or counteracting genes and pathways. Endosperm protein, however, is of low nutritional value due mainly to the high expression of the α-zein gene family, which encodes lysine-free proteins. Reduced levels of these proteins in the* opaque 2* (*o2*) mutant and α-zein RNAi (RNA interference) transgenic seed is compensated by an increase of non-zein proteins, leading to the rebalancing of the nitrogen sink and producing more or less constant levels of total proteins in the seed. The same rebalancing of zeins and non-zeins has been observed for maize seeds bred for 30% protein. In contrast to the nitrogen sink, storage of sulfur is controlled through the accumulation of specialized sulfur-rich proteins in maize endosperm. Silencing the synthesis of α-zeins through RNAi fails to raise sulfur-rich proteins. Although overexpression of the methionine-rich δ-zein can increase the methionine level in seeds, it occurs at least in part at the expense of the cysteine-rich β- and γ-zeins, demonstrating a balance between cysteine and methionine in sulfur storage. Therefore, we propose that the throttle for the flow of sulfur is placed before the synthesis of sulfur amino acids when sulfur is taken up and reduced during photosynthesis.

## STORAGE PROTEINS IN MAIZE SEED

Angiosperm seeds result from double fertilization and are usually the primary mode of reproduction. Besides their vital biological function, seeds are the most frequently harvested organs in agriculture. Therefore, their production has tremendous economic importance for humans and livestock. For this reason, maize has become one of the most productive cereal crops in the world in respect to yield per acreage. Maize seeds mainly consist of endosperm and embryo, which account for 90 and 10%, respectively, of the whole dry seed weight (Flint-Garcia et al., 2009). Starch and protein are mainly stored in endosperm, whereas most of the oil accumulates in the embryo.

**FIGURE 1:**
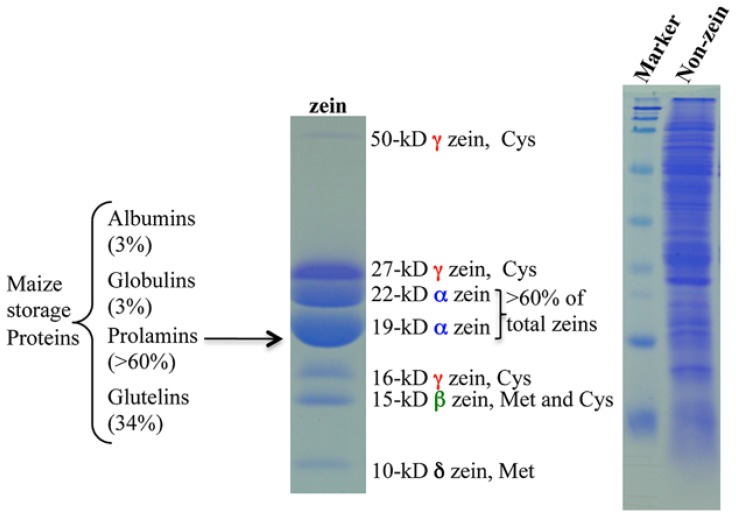
**Maize storage proteins. Prolamin proteins in maize are called zeins and the others are all classified as non-zeins**. Adapted from Wu et al. (2012).

Maize seeds contain ~10% proteins and ~70% of them are classified as storage proteins (Flint-Garcia et al., 2009). Based on their solubility in different solvents, endosperm proteins are divided into four groups: albumins, globulins, glutamines, and prolamins. The latter, called zeins, make up > 60% of total proteins (**Figure 1**). Zeins can be divided into four subfamilies, α (19 and 22 kDa), γ (50, 27, and 16 kDa), β (15 kDa), and δ (18 and 10 kDa; Esen, 1987; Coleman and Larkins, 1998; **Figure 1**). A common feature of all prolamins is internal tandem variable repeats of blocks of amino acids with primarily proline and glutamine, as first observed in a maize α-zein (Geraghty et al., 1981). Because of this feature, α-zeins lack essential amino acids like lysine, methionine, and tryptophan. Due to the high expression of α-zein genes in maize endosperm, the final levels of these three essential amino acids in total protein are very low (Osborne and Mendel, 1914). Therefore, maize cannot serve as a balanced dietary protein source for humans and monogastric animals and has to be supplemented with these amino acids, raising the cost of food supply worldwide (Mertz et al., 1964). Interestingly, the level of one amino acid, methionine, can reach sufficient levels in some cultivars, making supplements redundant (Messing and Fisher, 1991). The reason for this is that minor zeins, β, γ, and δ, have a high proportion of sulfur-rich amino acids and can vary in expression levels among maize cultivars. The δ-zeins are very rich in methionine, whereas the γ-zeins are abundant in cysteine; β-zein has high percentages of cysteine and methionine, while α-zeins lack both of them (Wu et al., 2012).

## PROTEOME BALANCING IN MAIZE ENDOSPERM

Maize domestication from its wild ancestor, teosinte, can be traced back to the Tehuacan Valley of Mexico as early as 8,000 years ago. During this process, teosinte underwent dramatic changes, not only in plant morphology, but also in seed composition (Flint-Garcia et al., 2009). Teosinte contains ~30% protein and has a high level of the methionine-rich δ-zeins (Swarup et al., 1995), but modern maize has only ~10% total protein and a low level of methionine-rich proteins in cultivars grown for consumption. Although one can achieve among all crops the highest yields of grain with maize, its protein level is much lower than soybean, which contains ~35% protein with sufficient lysine levels.

To investigate whether artificial selection can significantly change seed compositions, a long-term selection experiment has been carried out for more than one century at the University of Illinois. This has yielded four strains with substantially different protein levels: Illinois high protein (IHP), Illinois low protein (ILP), Illinois high protein reverse (IHPR), and Illinois low protein reverse (ILPR) with protein levels of 30, 4, 7, and 15%, respectively (Hopkins, 1899; Dudley and Lambert, 2004; Moose et al., 2004; Dudley, 2007). However, elevated protein levels are mainly due to zeins, which of course lack lysine (Osborne and Mendel, 1914). Therefore, IHP contains even relatively lower lysine levels (Lys_rel_) than those in normal maize (Wu and Messing, 2012).

An unexpected finding was when zein levels are lowered by the *opaque-2* mutation the relative lysine content was improved to a nearly sufficient level. This mutation affects an endosperm-specific transcription factor belonging to the bZIP family that is required for transactivation of several zein gene subfamilies (Schmidt et al., 1992; Cord Neto et al., 1995). The reduction in zein gene expression results in seeds with an opaque appearance. In the *o2* mutant, the main zein components, the α-zeins, are reduced by more than 60% in certain inbred lines. However, the total protein level remains almost unchanged by a compensatory increase of non-zein proteins with higher lysine levels (Holding and Larkins, 2009). As a consequence, the percentage of overall lysine is elevated. This compensation phenomenon indicates that nitrogen storage is controlled at the level of protein synthesis, leading to a more or less constant amount of total protein. However, as *o2* is recessive, pleiotropic and its penetration can vary in different α-zein haplotypes (Song and Messing, 2003), this trait requires two parental lines that are homozygous for *o2* and have additional QTLs for seed quality for hybrid seed production. Such QTLs, namely *o2* modifiers, are required to convert the starchy *o2* endosperm, which is unfavorable for storage and transport of large volumes of maize, to a hard kernel texture. This modified *o2* maize mutant is known as “Quality Protein Maize” or QPM (Vasal et al., 1980; Holding et al., 2008). Because of the loss of the opaque phenotype in QPM, it becomes difficult for breeders to maintain *o2* homozygosity through visual scoring. To simplify QPM breeding, high lysine maize lines can be created with RNA interference (RNAi) mutants, which reduce α-zein mRNA in a dominant and more targeted fashion. However, in the absence of *o2* modifiers, the resulting transgenic seeds also present an opaque phenotype (Segal et al., 2003; Huang et al., 2006; Wu and Messing, 2011).

**FIGURE 2:**
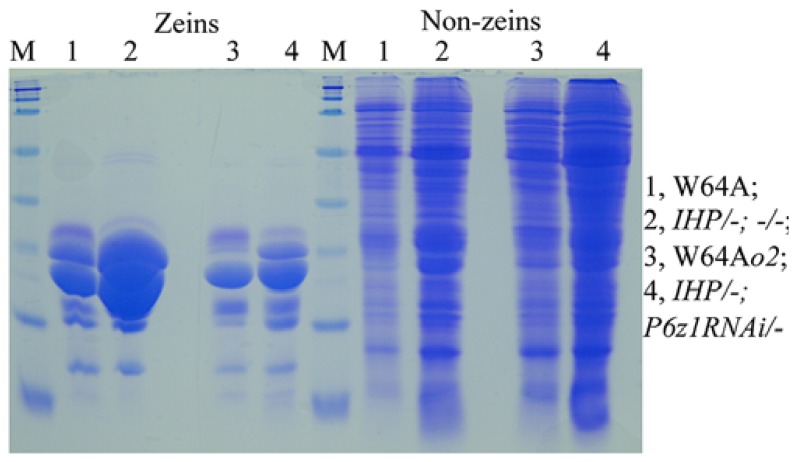
**Proteome rebalancing in *o2* mutant and IHP with suppressed α-zeins**. Lane 1 and 4, W64A and W64Ao2; lane 2 and 4, two F1 progeny of IHP × α-zeinRNAi/- not inheriting and inheriting the RNAi. Adapted from Wu and Messing (2012).

Although QPM has a hard endosperm and contains higher lysine than normal (Vasal et al., 1980), the total protein levels are still lower than in soybeans (Prasanna et al., 2001). If one could create maize lines that rival the nutritional quality of soybeans being high-protein and high-lysine, while having a hard-endosperm texture, one could investigate whether total protein could also be rebalanced by the mechanism operating in IHP. Indeed, when an α-zein RNAi event was crossed with IHP, the total protein level was maintained, although zeins were substantially reduced. Consequently, the non-zein fraction was dramatically increased to compensate for the loss of zeins (**Figure 2**). Moreover, suppression of zeins with an α-zein RNAi is incomplete, leaving a considerable amount of residual zeins that provide a hard vitreous endosperm texture without the need for *o2* modifiers (Wu and Messing, 2012).

What could be the mechanism underlying rebalancing the seed proteome? Sugars and amino acids produced during photosynthesis are transported to seeds for deposition as starch and protein. It seems that developing maize seeds possess compensatory mechanisms that sense protein content when zein synthesis is interrupted, leading to translation of other mRNAs instead of zein mRNAs. This transfer of ribosomes to a different mRNA pool could be as simple as mass action, or involve an intracellular signal transduction to attain a predetermined protein level. Such signal transduction would likely occur, although not exclusively, at the transcriptional, posttranscriptional or translational levels (Frizzi et al., 2010; Jia et al., 2013). No matter how proteome rebalancing operates to alter seed composition, breeders had to take a long-term selection approach to accumulate QTLs to regulate this tightly controlled program (Dudley and Lambert, 2004). When synthesis of soybean’s major storage proteins, glycinin and conglycinin, were suppressed in knockdown mutant lines, the seeds maintained nearly identical levels of total protein compared to the untransformed soybean cultivars, with similar seed size and weight (Schmidt et al., 2011), suggesting that proteome rebalancing in seeds might be a rather common event, providing a constant sink for reduced nitrogen during seed maturation. In addition, plant seeds seem to possess the ability to overcome a protein shortage by remodeling their protein composition for use during germination and early seeding growth. Profiling non-zein accumulation in *o2* and *α-zeinRNAi* mutants appears to follow two distinct patterns, with an overall slight increase of proteins in general and significant overexpression of several specific proteins (Holding and Larkins, 2009; Wu et al., 2012; Jia et al., 2013). Among the specifically enhanced expressed proteins, eIF2 and GAPDH have been identified as high-lysine containing proteins, which were thought to add a substantial contribution to the overall lysine elevation (Habben et al., 1993, 1995; Jia et al., 2013). However, what regulates their expression when zeins are suppressed remains unclear.

## SULFUR REBALANCING IN MAIZE ENDOSPERM

Sulfur amino acid deficiency differs from lysine deficiency in several ways. The essential amino acid methionine is the only amino acid that is currently chemically synthesized for supplementation of animal feed, because even soybean proteins do not provide sufficient levels in a dietary ration. However, in contrast to lysine, maize produces β- and δ-zein proteins that are very high in methionine residues. But in most maize inbreds, they just do not accumulate at sufficient levels although it has been shown that increased levels of sulfur in the soil can increase synthesis of sulfur-rich proteins in peas (Beach et al., 1985). Screens of seeds from different maize genetic backgrounds by germinating in the presence of lysine and threonine (LT) resulted in the discovery of one LT-resistant line (Phillips, 1985), where the δ-zein gene, rich in methionine codons, was overexpressed (Kirihara et al., 1988). It also was shown that this differential expression was subject to parental imprinting in hybrid crosses (Chaudhuri and Messing, 1994). This high δ-zein line was sufficient to replace synthetic methionine in a regular feed for chickens, with a direct impact on weight and feather quality (Messing and Fisher, 1991). Interestingly, the high expression of the δ-zein gene is not due to transcription, but rather to post-transcriptional regulation of its mRNA (Schickler, 1993). In fact, it appears that the regulation occurs via the un-translated regions (UTRs) of the mRNA, which was shown in transgenic seeds when the δ-zein mRNA UTRs were replaced by other sequences (Lai and Messing, 2002).

The allele-specific regulation of the methionine content, however, would unlikely be a pathway for rebalancing protein composition in seeds. In fact, high-lysine maize lines, like the *o2* mutant, where lysine-free α-zein proteins are reduced with a compensatory increase of other proteins, have failed to show any increase but rather somewhat of a decreased methionine level (Mertz et al., 1964; Phillips, 1985; Wu et al., 2012). This could also be an effect on the transcription of δ-zeins by O_2_ itself, as it also regulates another methionine-rich zein gene, the β-zein. Indeed, in knock-out mutants of δ-zeins in combination with a knock-down of β-zein, the accumulation of methionine is 40% less than that in normal maize lines (Wu et al., 2012). To eliminate the pleiotropic effects of O_2_ and only reduce the expression of α-zeins, methionine levels were also evaluated in the presence of α-zein RNAi. In this case, the methionine level did not increase along with lysine, showing that non-zein proteins are not as rich in sulfur amino acids as in lysine. Indeed, only 8% of the proteins in the maize protein database have methionine residues above 4%, while about 57% have lysine residues above 4% (Wu et al., 2012). Therefore, it appears that the sink for reduced nitrogen and sulfur operate differently during seed development.

**FIGURE 3:**
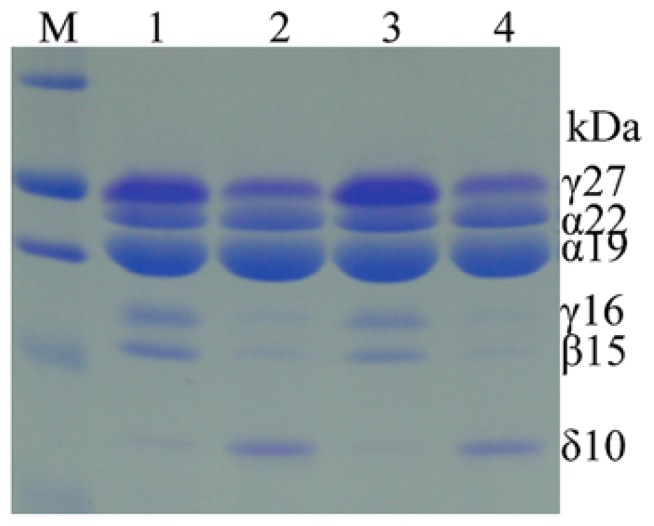
**Accumulation patterns of zeins in high-methionine transgenic and WT seeds**. Hi-Met transgenic seeds in lane 2 and 4 express much higher levels of the 10-kDa δ-zein, but lower levels of β- and γ-zeins than WT in lane 1 and 4. Adapted from Wu et al. (2012).

There is no apparent inferior kernel phenotype in high-methionine maize, in contrast to high-lysine α-zein RNAi maize, indicating that single gene manipulation could add a stable trait without agronomic compromises. Indeed, after several backcrosses of the chimeric δ-zein gene to a maize line that is low in methionine, overexpression of the 10-kDa zein gene remained stable (Lai and Messing, 2002). However, the transgenic line exhibited an interesting biochemical difference compared to normal maize (Wu et al., 2012). Unexpectedly, the accumulation of cysteine-rich γ- and β-zeins was dramatically suppressed (**Figure 3**). A recent study also found that selecting high-methionine variants from a maize population apparently resulted in low accumulation of the cysteine-rich 27-kDa γ-zein (Newell et al., 2014). These results suggest that increased methionine storage requires increased flow of reduced sulfur from cysteine to methionine, thereby reducing the translation of 27-kDa γ-zein mRNA. However, this shift could also be achieved with RNAi against γ- and β-zeins.

Perhaps, this could be explained as follows. Methionine and cysteine are the only two sulfur-containing amino acids among the twenty protein-containing L-amino acids. Sulfur is one of the essential elements for plant growth and is absorbed by root as sulfate (SO_4_^2-^) with an oxidation state +6. Sulfate has to be reduced to -2 through several enzymatic steps to form the intermediate product, cysteine. Because γ- and β-zeins are the cysteine-rich storage proteins, silencing their expression causes a significant reduction in cysteine level, indicating these proteins are the main sink for cysteine storage (Wu et al., 2012). If free cysteine is the first stable product with reduced sulfur, the majority of which is incorporated into cysteine-rich proteins, like γ-zeins and the excess would flow into methionine, its concentration would drive the translation of the δ- and β-zein (β-zein is rich both in cysteine and methionine) mRNA. Indeed, in RNAi against γ- and β-zeins, one can observe a boost in the accumulation of δ-zeins. Therefore, depriving the cysteine sink or increasing the methionine sink has the same result in that the flux of reduced sulfur flows through cysteine to methionine. This balance is made possible through the expression of different single/low copy number genes specialized for storage of these two amino acids (Wu et al., 2012).

**FIGURE 4:**
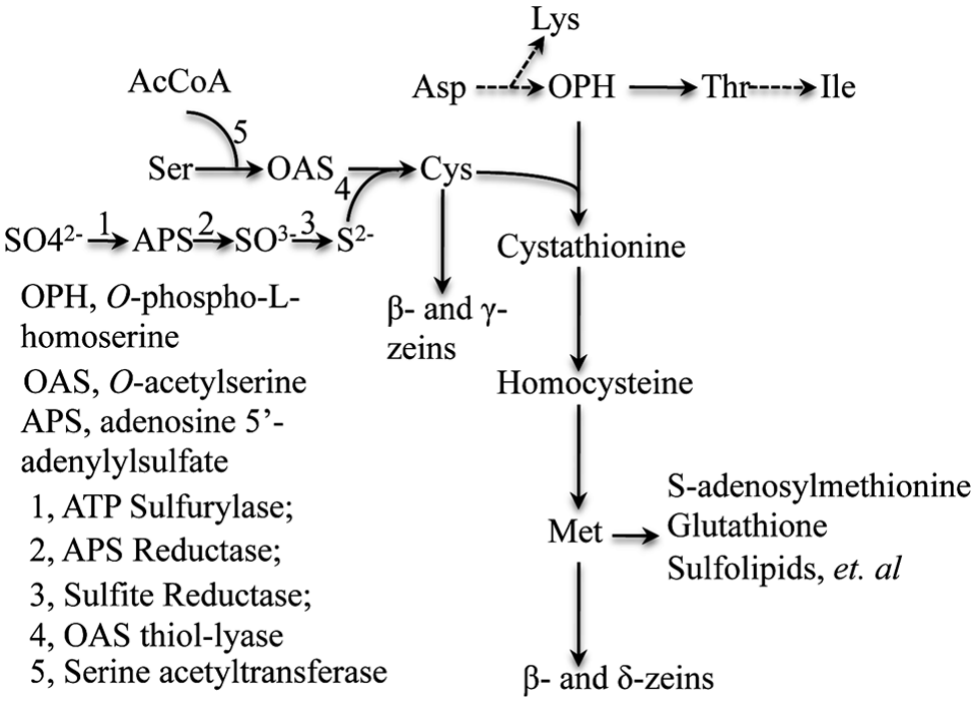
**Sulfate reduction and synthesis of cysteine and methionine pathways**. The flow of sulfur is shown to illustrate sink source relationship. Adapted from Wu et al. (2012).

Based on the above hypothesis, the major bottleneck for increasing seed methionine content is the capacity of sulfur absorption by roots and the efficiency by which sulfur can be reduced in the leaves during photosynthesis. Three enzymes ATP sulfurylase, APS reductase (APR) and sulfite reductase in this pathway combine coordinately to reduce sulfate with oxidation state +6 to sulfide with oxidation state -2 (**Figure 4**). Meanwhile, *O*-acetylserine (OAS), the other precursor for cysteine syhthesis, is formed from serine and acetyl-CoA catalyzed by serine acetyltransferase (SAT). And last, sulfide reacts with OAS, producing the end assimilation product of cysteine catalyzed by OAS thiol-lyase (Leustek et al., 2000). We propose such capacity of sulfur reduction could be enhanced by specific overexpression of the committing enzymes APR and SAT in leaf bundle sheath cells, where the sulfur reduction occurs and as a consequence improve cysteine and methionine sinks in seed.

## Conflict of Interest Statement

The authors declare that the research was conducted in the absence of any commercial or financial relationships that could be construed as a potential conflict of interest.
